# Queer in Chem: Q&A with Professor Nancy Williams

**DOI:** 10.1038/s42004-023-00970-x

**Published:** 2023-09-30

## Abstract

Nancy Scott Burke Williams is an Associate Professor of Chemistry at the Keck Science Department of Claremont McKenna, Pitzer, and Scripps Colleges in Claremont, California, where she has been in the faculty since 2003. She was born in Puyallup, WA to Burke and Nancy Williams, from whom she takes most of her names.

**Figure:**
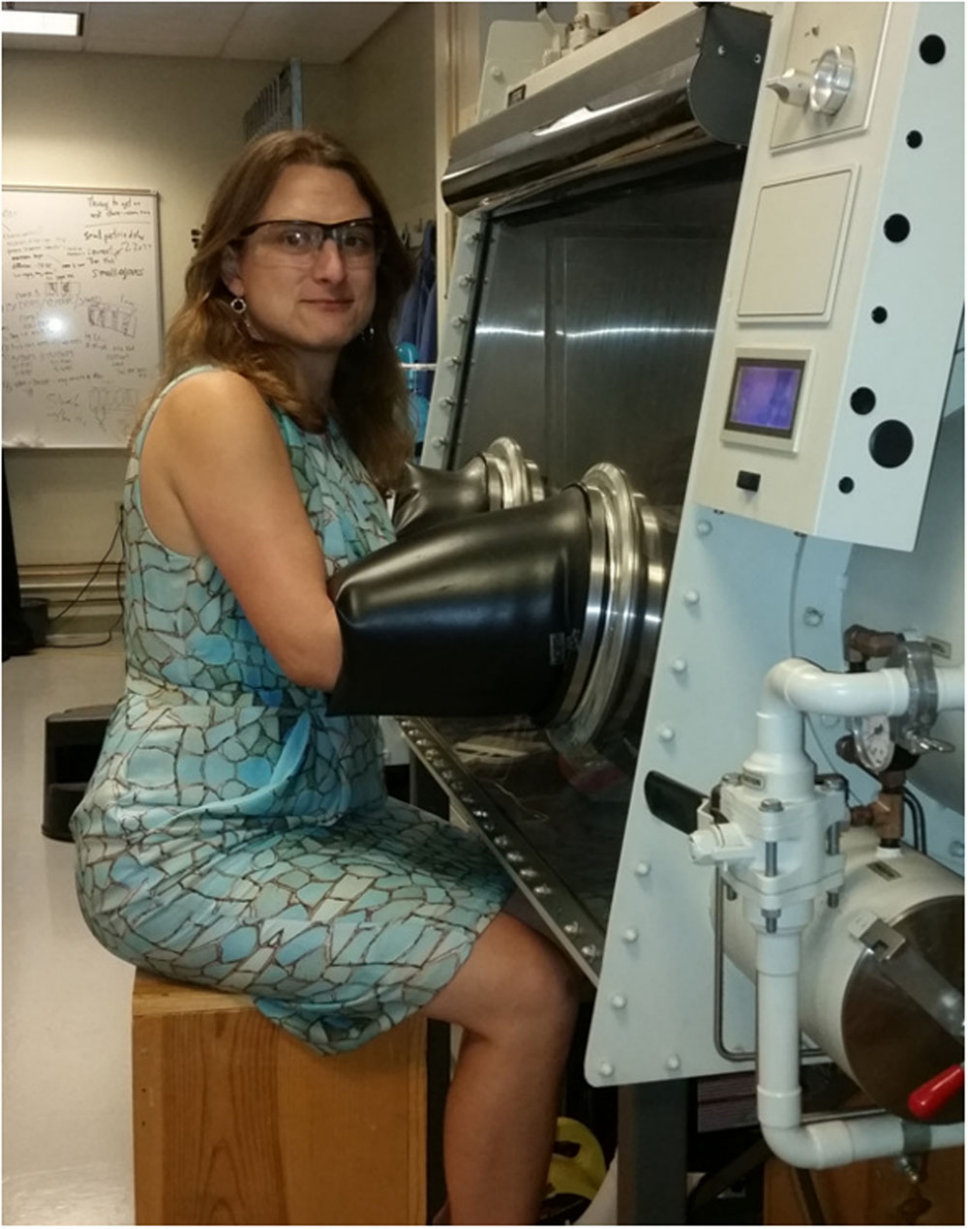
Nancy Williams

Nancy received her undergraduate degree from Harvey Mudd College under the research supervision of Mitsuru Kubota, and her PhD from Karen Goldberg (then at the University of Washington, now at Penn). She was a NATO-NSF postdoc with Gerard van Koten at the University of Utrecht in the Netherlands and did a second postdoc with M. S. Brookhart at UNC, Chapel Hill. Nancy came out as a queer, trans woman in 2013, and has been involved in a number of projects in the community, including volunteering with the Leadership LAB of the Los Angeles LGBT Center on Deep Canvassing from 2013 to 2017, serving on the organizational committee for the Resist March in 2017, Deep Canvassing with the New Conversation Initiative from 2018 to 2020, and singing with the Trans Chorus of Los Angeles from 2016 to 2022. She lives in Claremont with her spouse, Aithan, and her two cats, Atty and Gale. Her scientific interests are in late transition metals and the metal-ligand frameworks that support the making and breaking of strong, inert bonds such as those found in hydrocarbons.

Why did you choose to be a scientist?

I was encouraged to pursue science even from childhood, probably in no small part because I was assigned male at birth; I don’t think my cis sister got the same encouragement (but, to be fair, she also showed less interest). My father was a pharmacist and my mother had been a teacher—education and intellectual pursuits were highly valued in our family.

What direction do you think your research field should go in?

I think organic and inorganic chemistry have really diverged in their thinking about covalent bonding. In the world of organic chemistry, I think there’s an overreliance on some outdated forms of valence bond theory that frankly do a poor job of modeling reality. In the world of inorganic chemistry, I think we have so thoroughly embraced a molecular orbital approach that we have thrown the baby out with the bathwater. It’s possible to couple the power and simplicity of valence bond approaches with the accuracy and theoretical robustness of molecular orbital theory. We should be making better use of approaches like natural bond orbitals to develop a better understanding of chemical bonding throughout the periodic table that attempt to draw the insights from both traditions.

How does your queer or trans identity intersect with your identity as a scientist?

First and foremost, it has given me a deep understanding of the importance of Diversity, Equity, Inclusion and Justice work in science. I really deeply believe that science is for everyone, and that we have so many structures in place that prevent that from being true. The tax on time and energy that comes with marginalization are something I did not appreciate the magnitude of prior to coming out, and there is so much value in a scientific community that embraces everyone capable of loving a life of scientific discovery. There is a much bigger gap, for example, in my research productivity on sabbatical than during a teaching year than was the case before coming out; it’s because I’m not doing nearly as much of the invisible labor of being queer and trans. You might say that I have the option of not doing that invisible labor, but I don’t feel I do. I am constitutionally incapable of saying, “I’ve got mine, Jack” and not constantly working to make my surroundings more equitable. To understand inequity is to be bound to working the problem; I have to be the scientist I needed when I was closeted and afraid.

I also have a better understanding of how different life experiences can shape the kinds of questions we ask, the approaches we take, and the way we educate and train budding scientists.

Why do you think it is important to feel comfortable enough to bring your whole self to work?

It’s the difference between being able to throw yourself completely into the scientific endeavor and treating it as a “job”. Chemistry *is* a job, of course…I’d be very disappointed if I didn’t get a paycheck. But, as a teacher and practitioner of chemistry, my daily experience is that of a deeply rewarding calling. There’s a bit of a Maslow’s hierarchy of needs thing going on—if your whole self isn’t at work, it’s almost impossible to sustain that immersion in scientific research or education as the sacred endeavor that they are. I can still run a reaction or teach a class, but my energies are focused on protecting my sacred self, making my teaching and research mundane. I’m sure my teaching was *fine* during the period in which I was coming out, transitioning, and finding my feet, but I’m also sure that I didn’t go into this work to do *adequate* teaching, and that my students deserve better. They deserve *superlative* teaching, and that comes from not paying that daily “tax” of trying to fully exist and thrive even before I hit the classroom.

Have you faced any challenging situations in your professional life as a result of your queer or trans identity? What did you or others learn from these experiences?

Well, there was certainly a time when I didn’t think it was possible to be out and still be employed in science, and there was a period in which certain meetings and conferences felt *de facto* off-limits to people like me. Fortunately, there has also been a period in which that didn’t seem true, but unfortunately, those days are back again. I simply can’t or won’t travel to places that are overly restrictive of my civil rights or those of other chemists.

How can individual scientists support and celebrate their LGBTQ+ colleagues?

Above all else, I think it’s important in this moment for scientists to stand up to the rising oppression of minorities and marginalized groups in the US and other nations such as the UK. These hostile political environments are in open conflict with the Open Society that a vibrant scientific community requires. The current threats to the ability of children to read expansively, of institutions to protect scientists and other intellectuals from authoritarian interference, the hostile response to DEIJ efforts in our institutions and occasionally published in journals are all daggers pointed at the heart of the Open Society that thinkers like Karl Popper advocated for after the Second World War. These oppressive ideologues ape the desire for open-minded discourse and the importance of allowing people to ask difficult and unpopular questions, but they advocate for the toleration of intolerance, the platforming and elevation of hatred, and they often use openly eliminationist rhetoric, these days mostly focused at trans and non-binary people. Our cisgender and straight allies need to recognize this hatemongering for what it is, and to deprive it of the respectability and oxygen that it so desperately craves. We need our straight and cisgender allies to speak their support for diversity, equity, inclusion, and justice with their *chests*, and to not merely passively accept queer and trans inclusion in science, but to vocally celebrate it.

What action(s) do you feel employers in chemical research should take to make a difference for LGBTQ+ scientists?

We need much more effective support for those who are targeted by sexual harassment and violence in science. This is one place where employers are absolutely in the driver’s seat, and where their unwillingness to hold people to account has been front and center in the fight for equity. Far too often, employers choose the abusive scientist over the scientists who have been abused. Next, we need employers to show some backbone. Pride began when (mostly Black and Brown) trans and queer people stood up to cops with nightsticks. Too many employers think Pride means distributing a rainbow-colored pad of sticky notes every June instead of vocally supporting queer and especially trans and non-binary people when it gets hard. How many systems in your company aren’t built with trans people in mind? In how many places do your computer systems lack a non-binary option? Why hasn’t fixing that been a priority? How many queer people work for the company? Do you talk about that when you talk about diversity? Do non-binary people have an inclusive place to use the bathroom at work, where they are both safe and affirmed in who they are, or is simply using the bathroom a daily humiliation? Do trans women have anyone they can see as a “possibility model” that lets them see where they can go in the institution? Does your insurance cover gender affirming therapy?

*This interview was conducted by the editors of Communications Chemistry.*

